# Discrimination of *Bacillus subtilis* from Other *Bacillus* Species Using Specific Oligonucleotide Primers for the Pyruvate Carboxylase and Shikimate Dehydrogenase Genes

**DOI:** 10.4014/jmb.2205.05014

**Published:** 2022-07-08

**Authors:** Gawon Lee, Sojeong Heo, Tao Kim, Hong-Eun Na, Junghyun Park, Eungyo Lee, Jong-Hoon Lee, Do-Won Jeong

**Affiliations:** 1 Department of Food and Nutrition, Dongduk Women’s University, Seoul 02748, Republic of Korea; 2 Department of Food Science and Biotechnology, Kyonggi University, Suwon 16227, Republic of Korea

**Keywords:** *Bacillus subtilis*, 16S rRNA gene, multilocus sequence typing, *pyrA*, *aroE*

## Abstract

*Bacillus subtilis* is a useful bacterium in the food industry with applications as a starter strain for fermented food and as a probiotic. However, it is difficult to discriminate *B. subtilis* from other *Bacillus* species because of high phenotypic and genetic similarity. In this study, we employed five previously constructed multilocus sequence typing (MLST) methods for the discrimination of *B. subtilis* from other *Bacillus* species and all five MLST assays clearly distinguished *B. subtilis*. Additionally, the 17 housekeeping genes used in the five MLST assays also clearly distinguished *B. subtilis*. The pyruvate carboxylase (*pyrA*) and shikimate dehydrogenase (*aroE*) genes were selected for the discrimination of *B. subtilis* because of their high number of polymorphic sites and the fact that they displayed the lowest homology among the 17 housekeeping genes. Specific primer sets for the *pyrA* and *aroE* genes were designed and PCR products were specifically amplified from *B. subtilis*, demonstrating the high specificity of the two housekeeping genes for *B. subtilis*. This species-specific PCR method provides a quick, simple, powerful, and reliable alternative to conventional methods in the detection and identification of *B. subtilis*.

## Introduction

*Bacillus subtilis* is a spore-forming bacterium that can withstand a range of extreme environmental conditions [1]. *B. subtilis* has been detected in diverse habitats such as soil, air, and within plants [1]. Its spore-forming properties also permit entrance into the gastrointestinal tract of animals, where it can form vegetative cells from spores, thereby sporulating again [2, 3]. Thus, research into the application of *B. subtilis* in vaccine delivery into the gastrointestinal tract or as a probiotic has been conducted [4-6] .

*B. subtilis* has been detected in several types of fermented soybeans in East Asia, such as *meju* and *doenjang* in Korea, *douchi* in China, and *natto* and *miso* in Japan [7-11]. *B. subtilis* exhibits extracellular amylase and protease activities [12, 13]. These activities influence the production of amino acids and flavor compounds during soybean fermentation [14-16]. It is well known that these enzymatic activities contribute toward the quality and sensory properties of fermented soybeans [14, 17]. *B. subtilis* also produces several bacteriocins [18] and has therefore been used as a starter culture for soybean fermentation [19], as well as a commercial fungicide (Taegro; *B. subtilis* var. *amyloliquefaciens* strain FZB24; Novozymes, Denmark).

*B. subtilis* is generally regarded as a safe bacterium because of its long history of use in the food industry. It also produces several industrially-important enzymes such as xylanase, lichenase, cellulose, and pectinase. These enzymes, produced from non-genetically-modified *B. subtilis*, can be applied in the food industry [20]. Although much research into the commercial value of *B. subtilis* has been conducted, including applications in the food industry and in vaccine development, [5, 20], studies on methods to distinguish *B. subtilis* from other *Bacillus* species are lacking and most of them are identified methods after DNA purification such as restriction fragment length polymorphism or randomly amplified polymorphic DNA analysis [21-24]. For the commercial use of *B. subtilis*, it is necessary to ensure the specific isolation of this species from other related species. In this study, we developed a method to specifically distinguish *B. subtilis* and thereby ensure its purity as a resource.

## Materials and Methods

### Culture Conditions of *Bacillus* Species

*Bacillus* species were cultured in Tryptic Soy Agar (TSA; Difco, USA) and Tryptic Soy Broth (TSB; Difco) at 37°C for 18 h to ensure that the traits of this organism were maintained.

### Biochemical Characterization of *Bacillus* Species

*Bacillus* species were characterized biochemically using a commercially available API 50 CHB/E system according to the manufacturer's instructions (BioMérieux, France). For the biochemical analysis, strains were incubated in TSB at 37°C for 18 h according to the manufacturer's instructions and adjusted to an optical density (OD_600_) of 0.6. The bacterial suspension was added to API 50 CHB/E medium 1% (w/v), inoculated onto a API 50CH strip, and then incubated under aerobic conditions at 37°C for 24 and 48 h. The phenol red indicator ensures that the strip turns yellow when acid is produced by fermentation using the carbohydrates added to the strip during incubation. Finally, the results were analyzed using the online software apiweb (https://apiweb.biomerieux.com) by submitting negative and positive responses according to the reference color reading table.

### Comparative Genomics of *Bacillus* Species

For comparative genomic analysis of closely related *Bacillus* species, the genome sequence data of six *B. subtilis*, three *Bacillus siamensis*, five *Bacillus velezensis*, four *Bacillus amyloliquefaciens*, and three *Bacillus atrophaeus* strains were obtained from the NCBI database (http://ncbi.nlm.nih.gov/genomes) (Table 1). Phylogenetic analyses of the 16S rRNA gene, housekeeping genes, and multilocus sequence typing (MLST) sequences were performed using the maximum likelihood algorithm of the MEGA 7.0 software. The number of alleles and polymorphic sites, the discriminatory power (DP), and the typing efficiency (TE) of these housekeeping genes were analyzed using MLSTest software (http://www.ipe.unsa.edu.ar/software). TE is defined as the number of genotypes per polymorphic site for each housekeeping gene [25]. DP is the likelihood that two strains differentiate when randomly selected from a population of unrelated strains [25]. The number of non-synonymous (dN) and synonymous (dS) nucleotide substitutions per site was estimated using MEGA 7.0 software [26].

### Application of Species Particular Oligonucleotide Primer

To differentiate *B. subtilis* from other *Bacillus* species, two genes, *aroE* and *pycA*, were selected based on MLST. *B. subtilis*-specific primer sets were designed (Table 2). Genomic DNA of *Bacillus* species was extracted using a DNeasy tissue kit (Qiagen, Germany). Amplification of the *aroE* and *pycA* genes was performed using the primer sets aroE-F/-R and pycA-F/-R, respectively. The PCR conditions were as follows: an initial denaturation step at 95°C for 5 min, followed by 30 cycles consisting of 95°C for 30 sec, 60°C for 30 sec, and 72°C for 30 sec, then a post-extension step at 72°C for 5 min, and finally holding at 16°C in a T3000 Thermocycler (Biometra, Germany). Amplified PCR products were migrated on a 1.5% agarose gel.

## Results and Discussion

### Comparison of the 16S rRNA Sequence of *B. subtilis* with those of other *Bacillus* Species

The entire 16S rRNA gene sequence of *B. subtilis* KCCM 32835^T^ showed >99.1% similarity with the corresponding sequences from *B. amyloliquefaciens*, *B. siamensis*, *B. velezensis*, and *B. atrophaeus* (Table 1). There were 0–3 polymorphic sites in this gene sequence among *B. subtilis* strains, 3–7 polymorphic sites among *B. velezensis* strains (Tables 1 and S1), and 11–13 polymorphic sites among *B. atrophaeus* strains showing 99.1%–99.2% similarity (Tables 1 and S1). High similarity and the low number of polymorphic sites within the 16S rRNA gene among *Bacillus* species have led to misidentification when classifying *B. subtilis* [27, 28]. For this reason, these five *Bacillus* species cannot be clearly distinguished based on the 16S rRNA gene alone.

### Biochemical Characterization of *B. subtilis* and other *Bacillus* Species

To biochemically identify *B. subtilis*, the API 50 CHB/E system is recommended. However, using the API identification table, *B. subtilis* and *B. amyloliquefaciens* presented together and could not be distinguished, and the other three species analyzed (*i.e.*, *B. siamensis*, *B. velezensis*, and *B. atrophaeus*) were not presented. This may be a result of insufficient API data on these species or difficulties with classifying these particular species into API 50 CHB system.

In this experiments, none of the species used erythritol, D-arabinose, L-xylose, D-adonitol, methyl-BD-xylopyranoside, D-galactose, L-sorbose, rhamnose, dulcitol, α-methyl-D-mannoside, melezitose, xylitol, D-turanose, D-lyxose, D-tagatose, D-fucose, L-fucose, D-arabitol, L-arabitol, gluconate, 2-keto-gluconate, or 5-keto-gluconate, but all species used ribose, D-glucose, D-fructose, mannitol, sorbitol, α-methyl-D-glucoside, amygdalin, esculin, salicin, cellobiose, maltose, sucrose, raffinose, and starch. Overall, *B. subtilis* showed high substrate usability, while *B. amyloliquefaciens* showed low substrate usability. However, despite slight differences between strains, there were no clear differences between species (Table 3). These results suggested that the API 50 CHB/E biochemical assay is unable to accurately discriminate *B. subtilis* from other *Bacillus* species.

### Comparison of MLST Schemes for *B. subtilis*

**Fig. 1:**
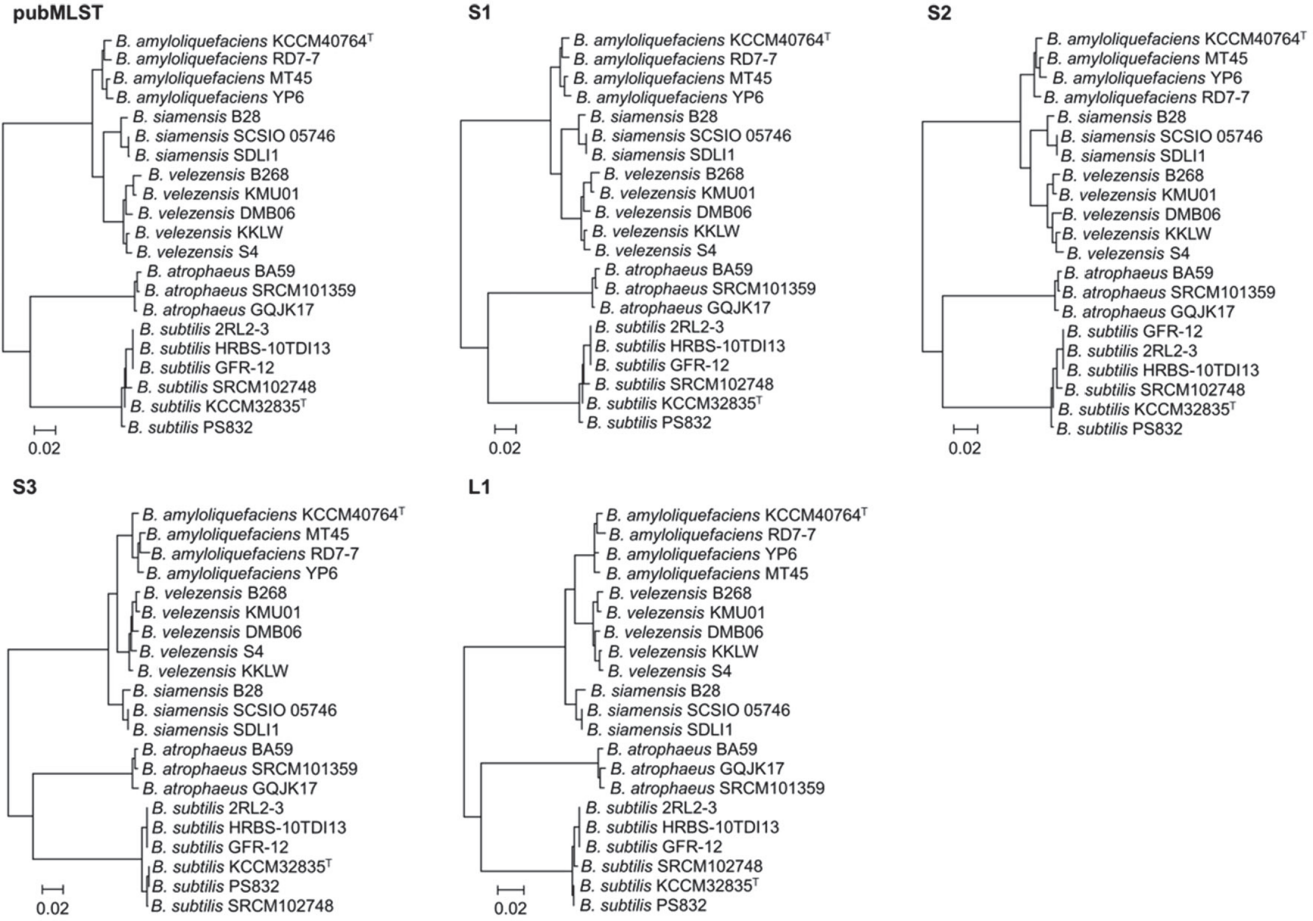
Phylogenetic analysis using five multilocus sequencing typing schemes. Data were compared using simple matching coefficients and were clustered by the maximum likelihood method. Branches with bootstrap values of 50% have been collapsed. The scale represents the pairwise distances expressed as the percentage of dissimilarity.

MLST is a useful approach for distinguishing bacterial species based on nucleotide sequences [29] and a public MLST scheme (pubMLST) for *B. subtilis* was developed using seven housekeeping genes [30] (Table 4). In addition, three further MLST schemes (S1–S3) for *B. subtilis* and one MLST scheme (L1) for *B. licheniformis* have been developed [30-34]. In the S1 scheme, the housekeeping gene is the same as that in the pubMLST, but the concatenated order is different [31]. The S2 scheme uses nine housekeeping genes, two more than in the pubMLST [32]. In all five MLST schemes, seven to nine housekeeping genes are used and all were able to distinguish *B. subtilis* from other *Bacillus* species on phylogenetic trees (Fig. 1). Indeed, the five MLST schemes showed >80.00% similarity between *B. subtilis* and other closely related *Bacillus* species. These results confirmed that MLST can more accurately distinguish between *Bacillus* species than the 16S rRNA gene sequences (Fig. 1 and Table S2).

Although the five MLST schemes were more discriminatory in terms of identifying *B. subtilis* from closely related *Bacillus* species, the analysis of seven or nine housekeeping genes is labor-intensive. Therefore, the contribution of each housekeeping gene in identifying *B. subtilis* from closely related *Bacillus* species was analyzed. The phylogenetic trees generated for each housekeeping gene were all able to clearly distinguish *B. subtilis* from other *Bacillus* species (Fig. S1).

The allelic variation was analyzed for each gene sequence and the number of polymorphic sites within each gene ranged from 78 (*adk*) to 1075 (*pycA*), and the number of allelic genes ranged from 11 (*adk*) to 19 (*pycA*) (Table 5). Although the number of polymorphic sites varied, the dN/dS ratio for each housekeeping gene showed no significant difference. The average dN/dS ratio across all MLST genes was 0.4044, and it was thereby assumed that these genes were not under positive selective pressure (*i.e.*, selection is against amino acid changes). For the *pycA* gene, the diversity in the amino acid sequence was lower compared with highly polymorphic sites. These findings were also evident in the TE (Table 5). In the five related *Bacillus* species analyzed, the TE of the 17 housekeeping genes ranged from 0.018 (*pycA*) to 0.141 (*adk*) (Table 5), whereas the DP did not differ significantly among these housekeeping genes, remaining at >0.935. These results suggested that the 17 housekeeping genes may be powerful markers for the discrimination of *B. subtilis* from other *Bacillus* species.

### Specific Oligonucleotide Primers for the Detection of *B. subtilis* by PCR

**Fig. 2:**
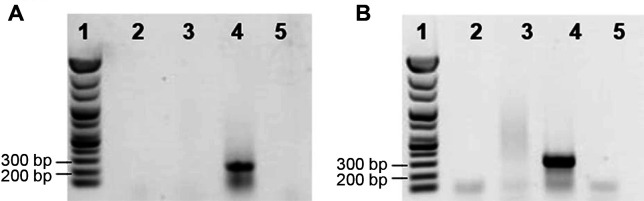
*Bacillus subtilis* species-specific PCR fragments of the *pycA* and *aroE* genes. A *pycA* gene, B *aroE* gene. Lane 1: 100 bp ladder; Lane 2: *Bacillus amyloliquefaciens* KCCM 40764^T^; Lane 3: *Bacillus siamensis* KCTC 13613^T^; Lane 4: *Bacillus subtilis* KCCM 32835^T^; Lane 5: *Bacillus velezensis* KCTC 13012^T^.

From the above results, it was confirmed that MLST and each of the housekeeping genes could distinguish *B. subtilis* from other *Bacillus* species. However, this method can only be applied after analyzing the nucleotide sequence of *B. subtilis* for isolation. Therefore, to more easily distinguish *B. subtilis*, a primer capable of identifying this species specifically was designed and its integrity was confirmed by PCR. Among the 17 housekeeping genes, *pycA* had the most alleles with 1075 polymorphic sites. The *pycA* nucleotide identity among *B. subtilis* strains was 98.9%–100%, compared with 79.7%–82.1% among other *Bacillus* species (Table S3). Therefore, we proposed that *pycA* was an appropriate gene to distinguish *B. subtilis* from other *Bacillus* species. Nucleotide sequences that could be distinguished were detected through comparative analysis, and a primer was designed to this sequence. PCR analysis confirmed amplification of *B. subtilis* DNA but not the DNA of other *Bacillus* species (Fig. 2).

The *aroE* gene sequence showed the lowest homology across strains among the 17 housekeeping genes. In *B. subtilis*, the *aroE* gene showed 98.8%–99.9% similarity among strains (Table S3). By contrast, other *Bacillus* species showed 67.0%–74.6% similarity in the *aroE* full sequence (Table S3). Hence, primers were designed against a partial sequence of the *aroE* gene, and it was confirmed that only *B. subtilis* DNA was amplified by PCR. In the above experiment, only two housekeeping genes, *pycA* and *aroE*, among 17 genes were applied to discriminate of *B. subtilis*. However as shown in table 5, we assumed that other 15 genes might also be possessed the potential for discrimination.

To assess the range of specificity of the PCR assay, the primer sets for the *pycA* and *aroE* genes were used in PCR analysis of 32 Bacillus strains, including eight *B. subtilis* strains. Amplicons for the *pycA* and *aroE* genes were only detected with *B. subtilis* strains (Fig. S2), and this assay may therefore have important implications for the accurate discrimination of *B. subtilis* from fermented food-derived *Bacillus* species.

As a result of the limitations of conventional approaches to *B. subtilis* identification, which include 16S rRNA gene sequence analysis and biochemical analysis, an auxiliary method was needed. MLST, and the housekeeping genes analyzed using this method, can clearly distinguish *B. subtilis* from other *Bacillus* species. In the current study, we showed that the *pycA* and *aroE* genes can be effectively used to screen for *B. subtilis* and clearly discriminate this species from other *Bacillus* species. These results confirmed that PCR amplification using our *B. subtilis*-specific primer set offers a quick, simple, powerful, and reliable method for accurately identifying *B. subtilis* from other *Bacillus* species.

## Supplemental Materials

Supplementary data for this paper are available on-line only at http://jmb.or.kr.

## Figures and Tables

**Table 1:** Bacillus strains for comparative genomic analysis and 16S rRNA homology.

Species	Strain	Accession No.	16S rRNA homology (%)	No. of polymorphic sites
*B. subtilis*	KCCM 32835^T*^	NZ_CP020102	100.0	(Reference strain)
*B. subtilis*	PS832	NZ_CP010053	100.0	0
*B. subtilis*	HRBS-10TDI13	NZ_CP015222	99.9	2
*B. subtilis*	GFR-12	NZ_CP032852	99.9	2
*B. subtilis*	2RL2-3	NZ_CP032857	99.8	3
*B. subtilis*	SRCM102748^*^	NZ_CP028212	99.8	3
*B. velezensis*	KMU01^*^	NZ_CP063768	99.8	3
*B. velezensis*	B268	NZ_CP053764	99.7	4
*B. velezensis*	S4	NZ_CP050424	99.7	4
*B. velezensis*	KKLW	NZ_CP054714	99.7	5
*B. velezensis*	DMB06^*^	NZ_CP083763	99.5	7
*B. velezensis*	KCTC 13012^T*^	-	-	-
*B. siamensis*	SCSIO 05746	NZ_CP025001	99.7	5
*B. siamensis*	SDLI1	NZ_CP013950.1	99.7	5
*B. siamensis*	B28^*^	NZ_CP066219	99.5	7
*B. siamensis*	KCTC 13613^T*^	-	-	-
*B. amyloliquefaciens*	MT45	NZ_CP011252	99.6	6
*B. amyloliquefaciens*	RD7-7	NZ_CP016913	99.6	6
*B. amyloliquefaciens*	YP6	NZ_CP032146	99.5	7
*B. amyloliquefaciens*	KCCM 40764^T*^	NC_014551	99.5	8
*B. amyloliquefaciens*	KCCM 12090^*^	-	-	-
*B. atrophaeus*	SRCM101359	NZ_CP021500	99.2	11
*B. atrophaeus*	GQJK17	NZ_CP022653	99.2	12
*B. atrophaeus*	BA59	NZ_CP024051	99.1	13

*Used for API and/or PCR analysis.

**Table 2:** Oligonucleotide primer sequences for the identification of *B. subtilis*.

Primer	Sequence (5'→3')	Expected size (bp)
pycA-F	GTC TTC CGT TCA GGA AAG GC	233
pycA-R	GAT CTC CCG TTT GGA TCG GCT C	
aroE-F	GGG GAA GGC TTC GTG AAG TC	278
*aroE*-R	CCC ACA GAC GTT GTA TGG ATG	

**Table 3:** Phenotypic characteristics of *Bacillus* species as analyzed by the API 50 CHB/E system.

Substrate	*B. subtilis*		*B. siamensis*		*B. velezensis*			*B. amyloliquefaciens*	
			
	KCCM 32835^T^	SRCM 102748	KCTC 13613^T^	B28	KMU01	KCTC 13012^T^	DMB06	KCCM 40764^T^	KCCM 12090
GLYcerol	+	+	+	+	+	+	-	w	+
L ARAbinose	+	+	+	+	+	+	+	-	-
D XYLose D-XYLose	+	w	+	+	+	+	+	-	-
D-MaNnosE	+	+	-	-	+	+	+	+	+
INOsitol	+	+	+	+	+	+	+	-	-
N-Acethyl-Glucosamine	w	-	+	-	+	+	-	+	+
ARButin	+	+	+	+	+	+	-	+	+
LACtose	w	w	w	+	+	+	+	+	-
MELibiose	+	+	-	-	-	-	+	-	-
TREhalose	+	+	-	-	+	+	+	-	-
INUlin	+	+	-	-	-	-	w	-	-
GLYcogen	+	+	+	+	+	+	+	-	-
GENtiobiose	w	w	-	-	-	+	-	w	w

Abbreviations: +: positive reaction; −: negative reaction; w: weak reaction (slight change).

**Table 4:** Five MLST methods for the analysis of *Bacillus* species.

Method	Concatenated order of genes for MLST	Target species	Reference
pubMLST	*glpF, ilvD, pta, purH, pycA, rpoD, tpiA*	*B. subtilis*	[30]
S1	*rpoD, glpF, ilvD, ptA, tpiA, pycA, purH*	*B. subtilis*	[31]
S2	*gyrA, gyrB, purH, glpF, pycA, ilvD, rpoD, tpiA, pta*	*B. subtilis*	[32]
S3	*gyrB, adk, pycA, pyrE, sucC, mutL, aroE*	*B. subtilis*	[33]
L1	*adk, ccpA, glpF, gmk, ilvD, pur, spo0A, tpi*	*B. paralicheniformis, B. licheniformis*	[34]

**Table 5:** Characteristics of housekeeping genes in 21 *B. subtilis* strains.

Housekeeping gene	Length (bp)	No. of alleles	No. of polymorphic sites	dN/dS	Typing efficiency (TE)	Discriminatory power (DP)
*adk*	654	11	78	0.4057	0.141	0.935
*aroE*	843	18	322	0.3909	0.056	0.983
*ccpA*	1005	16	269	0.4046	0.059	0.974
*glpF*	828	17	247	0.4122	0.069	0.978
*gmk*	615	15	136	0.4143	0.110	0.965
*gyrA*	2466	17	718	0.4098	0.024	0.978
*gyrB*	1917	17	532	0.4116	0.032	0.978
*ilvD*	1677	18	458	0.4057	0.039	0.983
*mutL*	1892	16	648	0.3972	0.025	0.970
*pta*	972	15	232	0.3973	0.065	0.965
*purH*	1539	17	428	0.4011	0.040	0.978
*pycA*	3450	19	1075	0.3990	0.018	0.991
*pyrE*	651	14	228	0.4015	0.061	0.961
*rpoD*	1122	16	218	0.4300	0.073	0.974
*spo0A*	804	16	187	0.4068	0.086	0.970
*sucC*	1158	15	219	0.4048	0.068	0.965
*tpiA*	762	16	114	0.3823	0.140	0.952
