# Supplementary material for: Discrimination of Bacillus subtilis from Other Bacillus Species Using Specific Oligonucleotide Primers for the Pyruvate Carboxylase and Shikimate Dehydrogenase Genes
Source: J Microbiol Biotechnol. 2022 Jul 8;32(8):1011–6. doi: 10.4014/jmb.2205.05014 (PMC9628935; doi:10.4014/jmb.2205.05014)
Supplement: Supplementary file 1 [file jmb-32-8-1011-supple.pdf]

https://doi.org/10.4014/jmb.2205.05014

Table S1. Polymorphic sites of 16S rRNA gene sequence in five *Bacillus* species.

Species	Strain	Nucleotide at polymorphic site (total 1550 bp)																							
		88	96	131	179	184	187	201	270	284	464	471	482	483	996	1015	1016	1029	1031	1044	1057	1250	1269	1460	1461
B. subtilis	KCCM 32835^T	C	T	G	G	T	A	A	C	G	G	G	T	A	G	A	T	A	G	G	T	A	A	T	A
B. subtilis	PS832	·	·	·	·	·	·	·	·	·	·	·	·	·	·	·	·	·	·	·	·	·	·	·	·
B. subtilis	HRBS-10TDI13	·	·	A	·	·	·	·	·	·	A	·	·	·	·	·	·	·	·	·	·	·	·	·	·
B. subtilis	GFR-12	·	·	A	·	·	·	·	·	·	A	·	·	·	·	·	·	·	·	·	·	·	·	·	·
B. subtilis	2RL2-3	·	·	A	·	·	·	·	·	·	A	·	·	·	·	·	·	·	·	-	·	·	·	·	·
B. subtilis	SRCM102748	·	·	·	·	·	·	·	T	A	A	·	·	·	·	·	·	·	·	·	·	·	·	·	·
B. velezensis	KMU01	·	·	·	·	·	·	G	·	·	·	A	C	·	·	·	·	·	·	·	·	·	·	·	·
B. velezensis	B268	·	·	·	·	·	·	G	·	A	·	A	C	·	·	·	·	·	·	·	·	·	·	·	·
B. velezensis	S4	·	·	·	·	C	.	G	·	·	·	A	C	·	·	·	·	·	·	·	·	·	·	·	·
B. velezensis	KKLW	·	·	·	·	C	G	G	·	·	·	A	C	·	·	·	·	·	·	·	·	·	·	·	·
B. velezensis	DMB06	·	·	·	·	·	·	G	·	·	.	A	C	·	A	·	·	·	·	·	·	G	·	A	T
B. siamensis	SCSIO05746	·	·	·	·	·	·	G	·	·	·	A	C	·	·	·	·	·	·	·	·	.	·	A	T
B. siamensis	SDLI1	·	·	·	·	·	·	G	·	·	·	A	C	·	·	·	·	·	·	·	·	.	·	A	T
B. siamensis	B28	·	·	·	C	·	·	G	·	·	·	A	C	·	·	·	·	·	·	·	·	G	·	A	T
B. amyloliquefaciens	MT45	·	·	·	C	·	·	G	·	·	·	A	C	·	·	·	·	·	·	·	·	G	·	·	T
B. amyloliquefaciens	RD7-7	·	·	·	C	·	·	G	·	·	·	A	C	·	·	·	·	·	·	·	·	G	·	·	T
B. amyloliquefaciens	YP6	·	C	·	C	·	·	.	·	·	·	A	C	G	·	·	·	·	·	·	·	G	·	·	T
B. amyloliquefaciens	KCCM 40764^T	T	·	·	C	·	·	G	·	·	·	A	C	·	·	·	·	·	·	·	C	G	·	·	T
B. atrophaeus	SRCM101359	·	·	·	C	·	·	·	T	A	·	.	C	·	·	C	C	G	T	·	·	·	G	A	T
B. atrophaeus	GQJK17	·	·	·	C	·	·	·	T	A	·	A	C	·	·	C	C	G	T	·	·	·	G	A	T
B. atrophaeus	BA59	·	·	·	C	·	·	·	T	A	·	A	C	·	·	C	C	G	T	-	·	·	G	A	T

Abbreviations: ·, same sequence with B. subtilis KCCM 32835^T; -, non-nucleotide.

1 **Table S2. Homology of concatenated sequence of each MLST method.**

Species	Strain	Homology (%)				
		pubMLST	S1	S2	S3	L1
B. subtilis	KCCM 32835[T]	100	100	100	100	100
B. subtilis	PS832	99.5	99.5	99.8	100	100
B. subtilis	HRBS-10TDI13	99.3	99.3	99.0	98.9	99.4
B. subtilis	2RL2-3	99.3	99.3	99.0	98.9	99.4
B. subtilis	GFR-12	99.3	99.3	99.0	98.9	99.4
B. subtilis	SRCM102748	99.4	99.4	99.4	99.8	99.5
B. velezensis	KMU01	82.0	82.0	82.1	80.3	84.5
B. velezensis	B268	81.7	81.7	82.1	80.3	84.4
B. velezensis	KKLW	82.0	82.0	82.2	80.4	84.8
B. velezensis	S4	82.1	82.1	82.1	80.3	84.6
B. velezensis	DMB06	81.6	81.6	82.1	80.4	84.7
B. siamensis	SCSIO 05746	82.1	82.1	82.2	80.0	84.9
B. siamensis	SDLI1	82.1	82.1	82.2	80.0	84.9
B. siamensis	B28	82.0	82.0	82.1	80.1	84.9
B. amyloliquefaciens	MT45	82.4	82.4	82.2	80.5	84.8
B. amyloliquefaciens	RD7-7	82.2	82.2	82.2	80.6	84.7
B. amyloliquefaciens	YP6	82.4	82.4	82.2	80.6	84.7
B. amyloliquefaciens	KCCM 40764[T]	82.1	82.1	82.1	80.2	84.5
B. atrophaeus	GQJK17	83.5	83.5	83.6	82.1	85.2
B. atrophaeus	SRCM101359	83.2	83.2	83.5	82.2	85.1
B. atrophaeus	BA59	83.2	83.2	83.5	82.2	85.1

Table S3. Similarity of housekeeping gene sequences in five *Bacillus* species.

		Homology (%)																
		Adk	*aroE*	*ccpA*	*glpF*	*gmk*	*gyrA*	*gyrB*	*ilvD*	*MutL*	*pta*	*purH*	*pycA*	*pyrE*	*rpoD*	*spo0A*	*sucC*	*tpiA*
B. subtilis	KCCM 32835[T]	100	100	100	100	100	100	100	100	100	100	100	100	100	100	100	100	100
B. subtilis	PS832	100	99.9	100	99.6	100	100	100	100	99.6	100	100	100	100	96.6	100	100	100
B. subtilis	HRBS-10TDI13	98.8	98.8	99.9	99.3	99.5	99.0	99.1	99.9	97.4	99.1	96.9	98.9	97.7	99.1	99.6	99.4	100
B. subtilis	GFR-12	98.8	98.8	99.9	99.3	99.5	99.0	99.1	99.9	97.4	99.1	96.9	98.9	97.7	99.1	99.6	99.4	100
B. subtilis	2RL2-3	98.8	98.8	99.9	99.3	99.5	99.0	99.1	99.9	97.4	99.1	96.9	98.9	97.7	99.1	99.6	99.4	100
B. subtilis	SRCM102748	100	99.4	99.5	98.8	100	99.0	99.2	99.0	99.6	100	98.8	99.9	100	99.5	100	100	99.7
B. velezensis	KMU01	91.1	68.1	83.1	77.3	83.6	79.0	80.3	81.2	74.8	82.9	81.0	79.8	73.9	84.9	85.3	87.9	92.8
B. velezensis	B268	91.1	68.2	83.1	77.4	84.1	79.2	80.3	81.2	74.8	83.1	81.0	79.8	73.9	84.9	85.2	87.9	92.4
B. velezensis	S4	91.1	67.3	82.8	77.8	83.6	78.9	80.2	81.0	74.9	83.2	81.2	79.7	73.7	85.0	85.4	88.0	93.2
B. velezensis	KKLW	91.1	68.3	83.3	77.9	83.6	79.2	80.5	80.8	74.5	83.4	81.2	79.8	74.0	84.7	85.7	87.9	93.3
B. velezensis	DMB06	91.1	68.0	83.2	78.0	84.1	78.9	80.4	80.8	75.1	82.4	81.1	79.8	73.7	84.8	85.4	88.0	93.2
B. siamensis	SCSIO 05746	90.5	68.0	82.4	77.8	85.5	79.2	79.8	81.6	74.4	83.7	80.8	79.9	74.3	84.6	85.3	88.2	92.8
B. siamensis	SDLI1	90.5	68.0	82.4	77.8	85.5	79.2	79.8	81.5	74.4	83.7	80.8	79.9	74.3	84.6	85.3	88.2	92.8
B. siamensis	B28	90.2	67.0	82.9	77.5	85.5	79.1	80.2	81.2	74.4	83.5	81.3	79.7	74.8	84.6	85.1	88.0	92.9
B. amyloliquefaciens	MT45	91.6	68.6	82.5	78.0	85.0	79.2	80.0	81.0	74.2	84.2	81.1	79.8	75.1	84.4	85.9	88.0	92.7
B. amyloliquefaciens	RD7-7	91.4	69.0	82.7	77.3	85.2	79.3	80.2	81.0	74.3	83.7	81.2	79.9	74.8	84.4	85.6	87.7	92.9
B. amyloliquefaciens	YP6	91.6	68.9	82.8	77.8	84.9	79.2	80.3	80.7	74.5	84.2	81.3	79.9	75.1	84.6	85.4	87.9	92.8
B. amyloliquefaciens	KCCM 40764[T]	91.3	68.1	82.3	77.8	84.7	79.3	80.1	80.6	74.4	83.6	81.2	79.7	75.0	84.4	85.6	88.2	92.7
B. atrophaeus	SRCM101359	93.1	74.6	79.9	83.6	86.8	79.5	80.8	82.3	76.1	85.6	82.5	81.8	77.4	86.8	84.1	88.1	90.3
B. atrophaeus	GQJK17	92.8	74.4	80.8	83.6	86.5	79.4	80.3	82.4	76.1	85.9	82.2	82.1	78.0	86.7	84.0	87.9	90.6
B. atrophaeus	BA59	93.1	74.5	80.4	83.6	86.8	79.2	80.6	82.2	76.1	85.5	82.4	81.9	77.7	86.6	84.1	87.7	90.4

Fig. S1. Phylogenetic tree of housekeeping gene used in the developed MLST method. The data were compared using simple matching coefficients and were clustered by the maximum likelihood method. Branches with bootstrap values of 50% have been collapsed. The scale the diagram is the pairwise distance expressed as the percentage of dissimilarity.

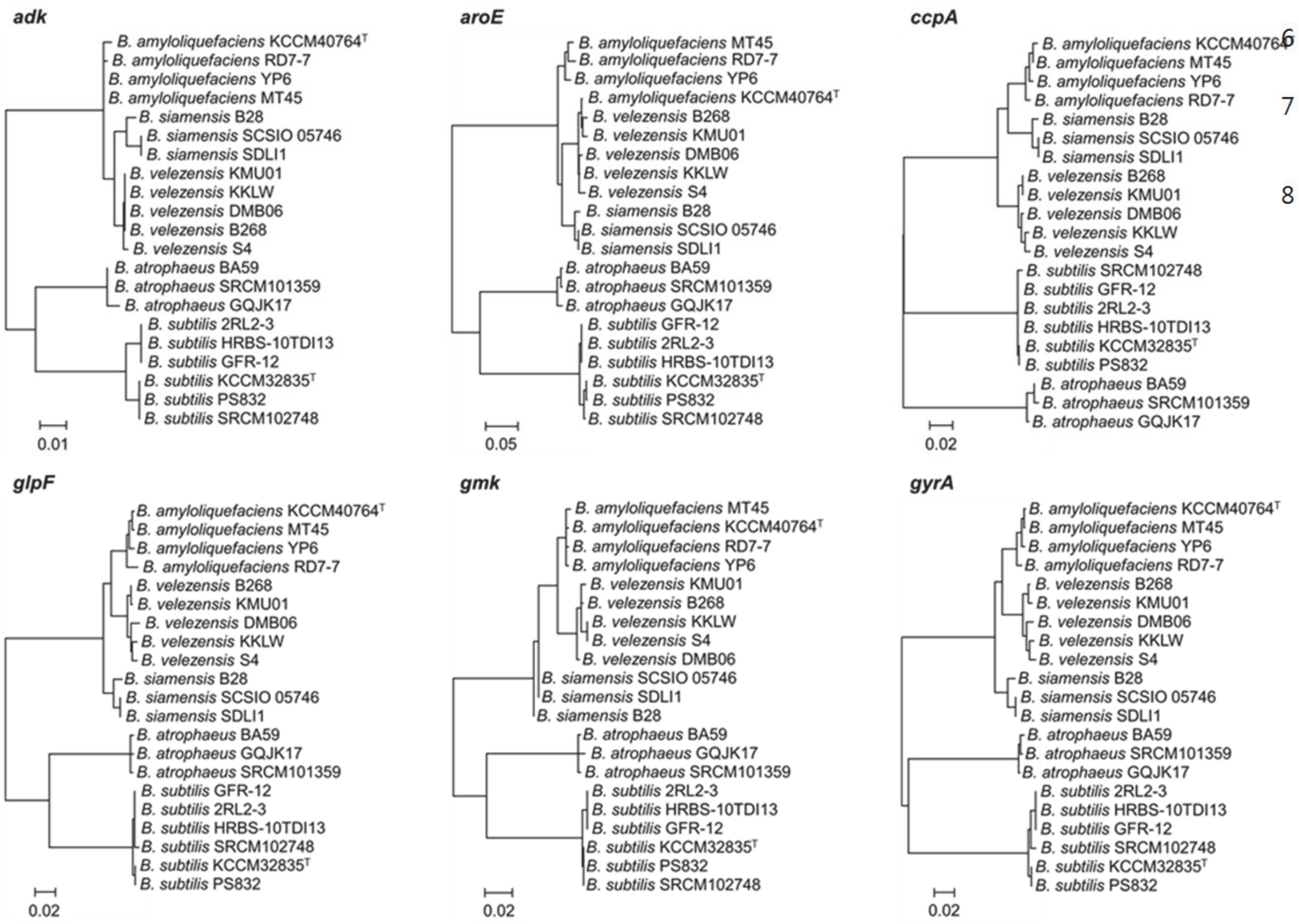

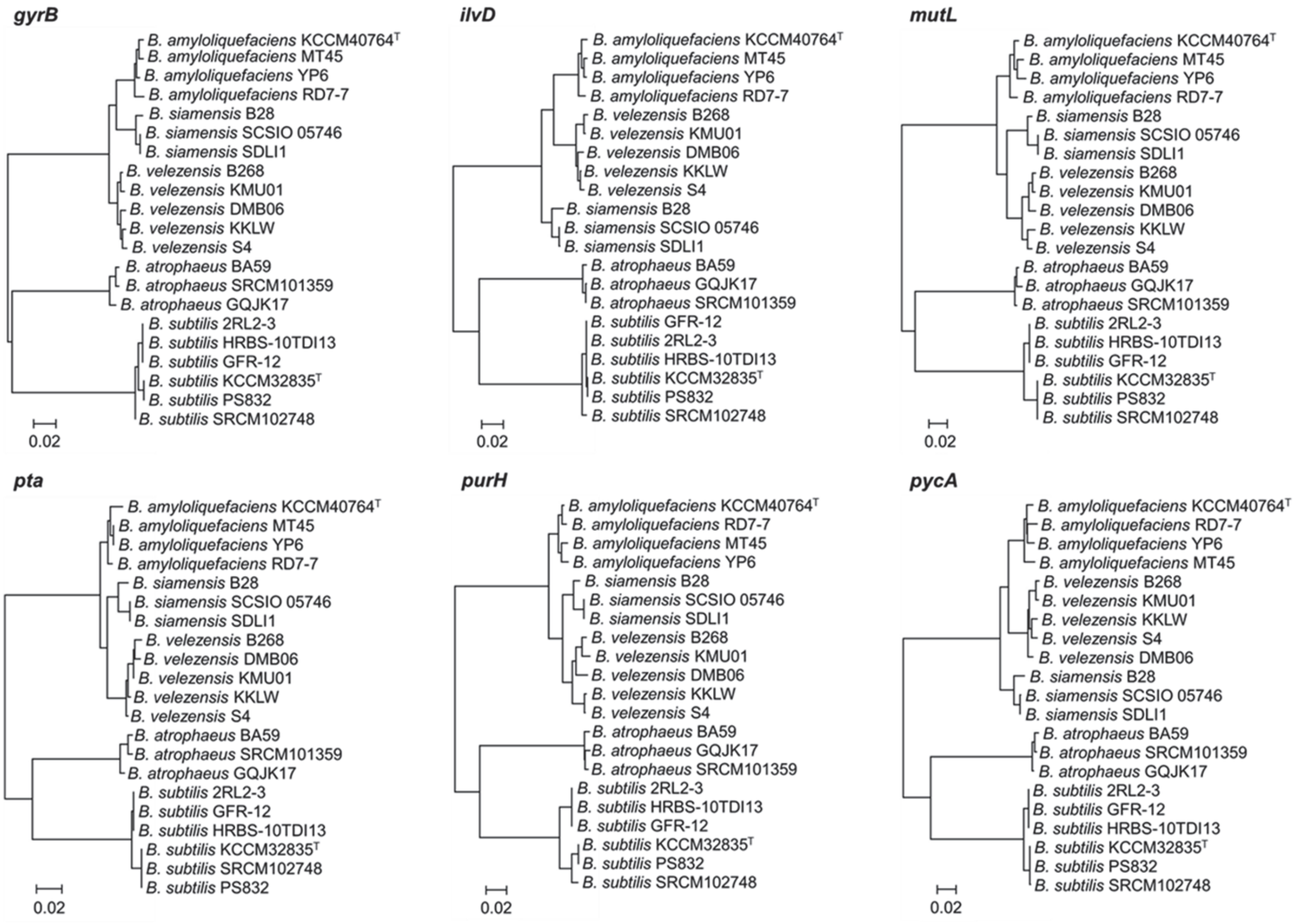
gyrB
B. amyloliquefaciens KCCM40764T
B. amyloliquefaciens MT45
B. amyloliquefaciens YP6
B. amyloliquefaciens RD7-7
B. siamensis B28
B. siamensis SCSIO 05746
B. siamensis SDLI1
B. velezensis B268
B. velezensis KMU01
B. velezensis DMB06
B. velezensis KKLW
B. velezensis S4
B. atrophaeus BA59
B. atrophaeus SRCM101359
B. atrophaeus GQJK17
B. subtilis 2RL2-3
B. subtilis HRBS-10TDI13
B. subtilis GFR-12
B. subtilis KCCM32835T
B. subtilis PS832
B. subtilis SRCM102748
0.02
ilvD
B. amyloliquefaciens KCCM40764T
B. amyloliquefaciens MT45
B. amyloliquefaciens YP6
B. amyloliquefaciens RD7-7
B. velezensis B268
B. velezensis KMU01
B. velezensis DMB06
B. velezensis KKLW
B. velezensis S4
B. siamensis B28
B. siamensis SCSIO 05746
B. siamensis SDLI1
B. atrophaeus BA59
B. atrophaeus GQJK17
B. atrophaeus SRCM101359
B. subtilis GFR-12
B. subtilis 2RL2-3
B. subtilis HRBS-10TDI13
B. subtilis KCCM32835T
B. subtilis PS832
B. subtilis SRCM102748
0.02
mutL
B. amyloliquefaciens KCCM40764T
B. amyloliquefaciens MT45
B. amyloliquefaciens YP6
B. amyloliquefaciens RD7-7
B. siamensis B28
B. siamensis SCSIO 05746
B. siamensis SDLI1
B. velezensis B268
B. velezensis KMU01
B. velezensis DMB06
B. velezensis KKLW
B. velezensis S4
B. atrophaeus BA59
B. atrophaeus GQJK17
B. atrophaeus SRCM101359
B. subtilis 2RL2-3
B. subtilis HRBS-10TDI13
B. subtilis GFR-12
B. subtilis KCCM32835T
B. subtilis PS832
B. subtilis SRCM102748
0.02
pta
B. amyloliquefaciens KCCM40764T
B. amyloliquefaciens MT45
B. amyloliquefaciens YP6
B. amyloliquefaciens RD7-7
B. siamensis B28
B. siamensis SCSIO 05746
B. siamensis SDLI1
B. velezensis B268
B. velezensis DMB06
B. velezensis KMU01
B. velezensis KKLW
B. velezensis S4
B. atrophaeus BA59
B. atrophaeus SRCM101359
B. atrophaeus GQJK17
B. subtilis 2RL2-3
B. subtilis GFR-12
B. subtilis HRBS-10TDI13
B. subtilis KCCM32835T
B. subtilis SRCM102748
B. subtilis PS832
0.02
purH
B. amyloliquefaciens KCCM40764T
B. amyloliquefaciens RD7-7
B. amyloliquefaciens MT45
B. amyloliquefaciens YP6
B. siamensis B28
B. siamensis SCSIO 05746
B. siamensis SDLI1
B. velezensis B268
B. velezensis KMU01
B. velezensis DMB06
B. velezensis KKLW
B. velezensis S4
B. atrophaeus BA59
B. atrophaeus GQJK17
B. atrophaeus SRCM101359
B. subtilis 2RL2-3
B. subtilis HRBS-10TDI13
B. subtilis GFR-12
B. subtilis KCCM32835T
B. subtilis PS832
B. subtilis SRCM102748
0.02
pycA
B. amyloliquefaciens KCCM40764T
B. amyloliquefaciens RD7-7
B. amyloliquefaciens YP6
B. amyloliquefaciens MT45
B. velezensis B268
B. velezensis KMU01
B. velezensis KKLW
B. velezensis S4
B. velezensis DMB06
B. siamensis B28
B. siamensis SCSIO 05746
B. siamensis SDLI1
B. atrophaeus BA59
B. atrophaeus SRCM101359
B. atrophaeus GQJK17
B. subtilis 2RL2-3
B. subtilis GFR-12
B. subtilis HRBS-10TDI13
B. subtilis KCCM32835T
B. subtilis PS832
B. subtilis SRCM102748
0.02

9

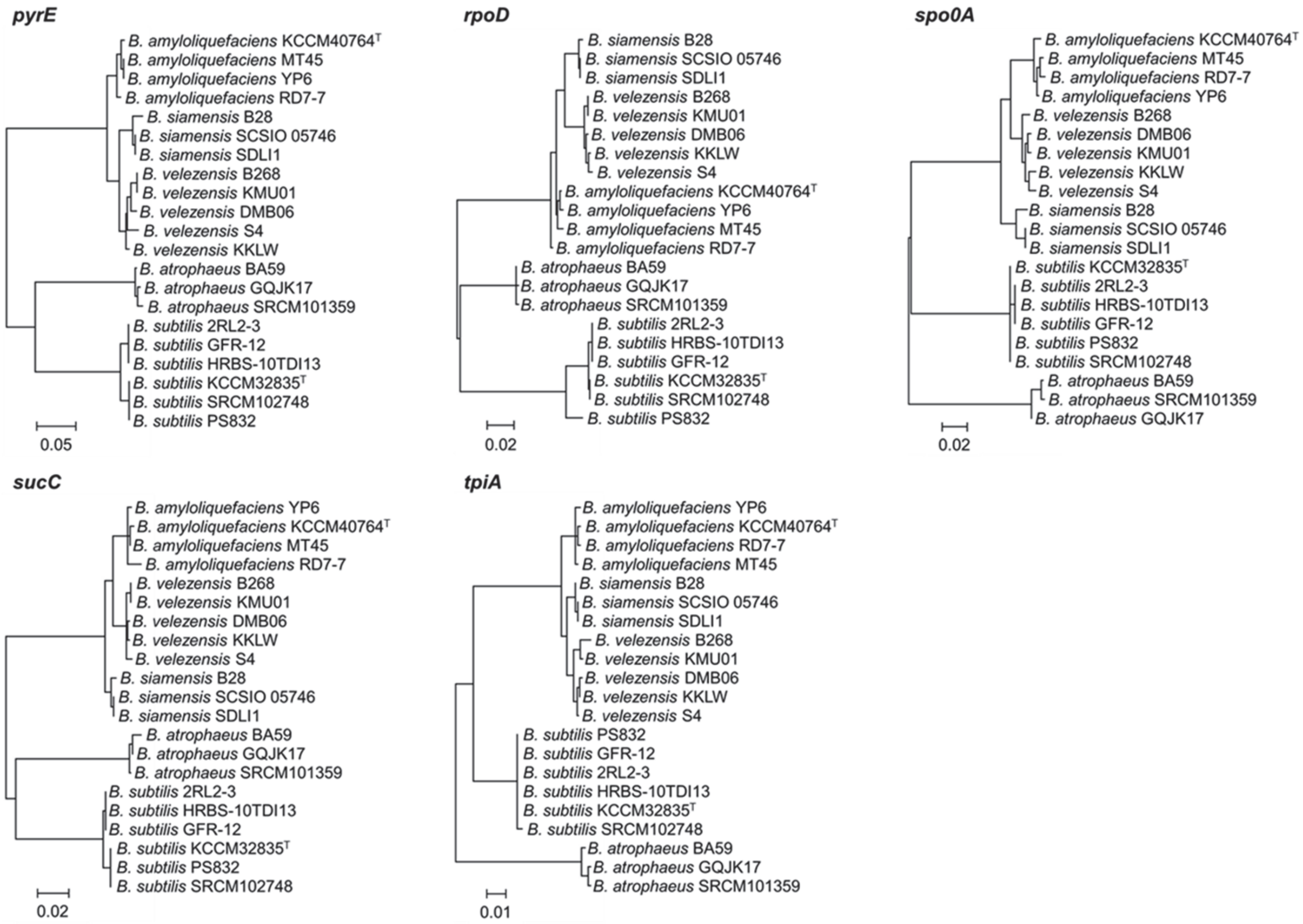

pyrE
B. amyloliquefaciens KCCM40764T
B. amyloliquefaciens MT45
B. amyloliquefaciens YP6
B. amyloliquefaciens RD7-7
B. siamensis B28
B. siamensis SCSIO 05746
B. siamensis SDLI1
B. velezensis B268
B. velezensis KMU01
B. velezensis DMB06
B. velezensis S4
B. velezensis KKLW
B. atrophaeus BA59
B. atrophaeus GQJK17
B. atrophaeus SRCM101359
B. subtilis 2RL2-3
B. subtilis GFR-12
B. subtilis HRBS-10TDI13
B. subtilis KCCM32835T
B. subtilis SRCM102748
B. subtilis PS832
0.05
rpoD
B. siamensis B28
B. siamensis SCSIO 05746
B. siamensis SDLI1
B. velezensis B268
B. velezensis KMU01
B. velezensis DMB06
B. velezensis KKLW
B. velezensis S4
B. amyloliquefaciens KCCM40764T
B. amyloliquefaciens YP6
B. amyloliquefaciens MT45
B. amyloliquefaciens RD7-7
B. atrophaeus BA59
B. atrophaeus GQJK17
B. atrophaeus SRCM101359
B. subtilis 2RL2-3
B. subtilis HRBS-10TDI13
B. subtilis GFR-12
B. subtilis KCCM32835T
B. subtilis SRCM102748
B. subtilis PS832
0.02
spo0A
B. amyloliquefaciens KCCM40764T
B. amyloliquefaciens MT45
B. amyloliquefaciens RD7-7
B. amyloliquefaciens YP6
B. velezensis B268
B. velezensis DMB06
B. velezensis KMU01
B. velezensis KKLW
B. velezensis S4
B. siamensis B28
B. siamensis SCSIO 05746
B. siamensis SDLI1
B. subtilis KCCM32835T
B. subtilis 2RL2-3
B. subtilis HRBS-10TDI13
B. subtilis GFR-12
B. subtilis PS832
B. subtilis SRCM102748
B. atrophaeus BA59
B. atrophaeus SRCM101359
B. atrophaeus GQJK17
0.02
sucC
B. amyloliquefaciens YP6
B. amyloliquefaciens KCCM40764T
B. amyloliquefaciens MT45
B. amyloliquefaciens RD7-7
B. velezensis B268
B. velezensis KMU01
B. velezensis DMB06
B. velezensis KKLW
B. velezensis S4
B. siamensis B28
B. siamensis SCSIO 05746
B. siamensis SDLI1
B. atrophaeus BA59
B. atrophaeus GQJK17
B. atrophaeus SRCM101359
B. subtilis 2RL2-3
B. subtilis HRBS-10TDI13
B. subtilis GFR-12
B. subtilis KCCM32835T
B. subtilis PS832
B. subtilis SRCM102748
0.02
tpiA
B. amyloliquefaciens YP6
B. amyloliquefaciens KCCM40764T
B. amyloliquefaciens RD7-7
B. amyloliquefaciens MT45
B. siamensis B28
B. siamensis SCSIO 05746
B. siamensis SDLI1
B. velezensis B268
B. velezensis KMU01
B. velezensis DMB06
B. velezensis KKLW
B. velezensis S4
B. subtilis PS832
B. subtilis GFR-12
B. subtilis 2RL2-3
B. subtilis HRBS-10TDI13
B. subtilis KCCM32835T
B. subtilis SRCM102748
B. atrophaeus BA59
B. atrophaeus GQJK17
B. atrophaeus SRCM101359
0.01

Fig. S2. Identification of *B. subtilis* using *B. subtilis* specific primer. (A) *pycA* gene, (B) *aroE* gene.

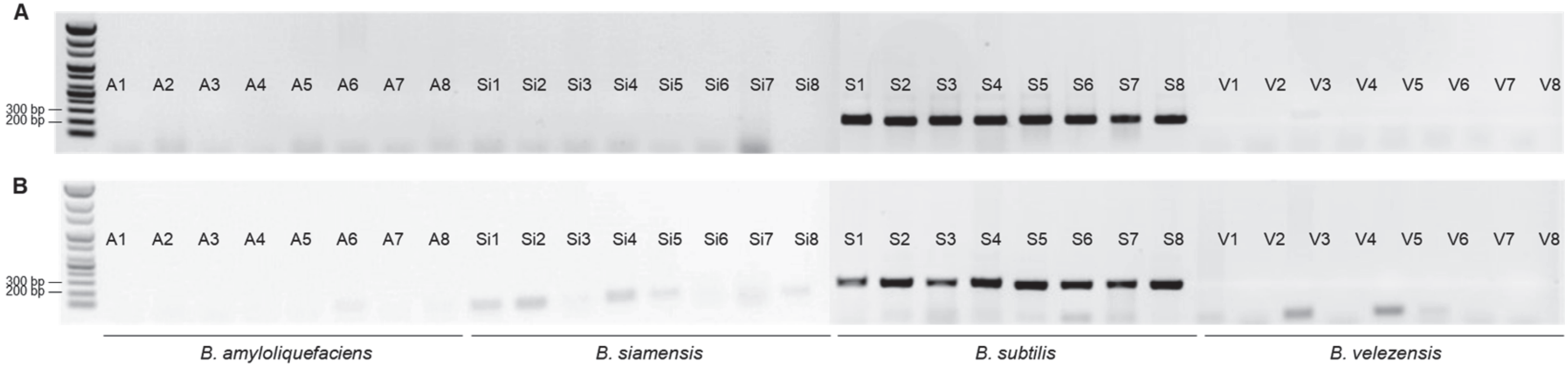

A1 *B. amyloliquefaciens* 0D3
A2 *B. amyloliquefaciens* 0M19
A3 *B. amyloliquefaciens* 0SSD26
A4 *B. amyloliquefaciens* 10SSD4
A5 *B. amyloliquefaciens* 7D4
A6 *B. amyloliquefaciens* 7SSD37
A7 *B. amyloliquefaciens* 7SSM29
A8 *B. amyloliquefaciens* GN16

Si1 *B. siamensis* 0M10
Si2 *B. siamensis* 0M17
Si3 *B. siamensis* 0SSD23
Si4 *B. siamensis* 7M3
Si5 *B. siamensis* 7SSD25
Si6 *B. siamensis* 7SSD28
Si7 *B. siamensis* 7SSM21
Si8 *B. siamensis* 7SSM37

S1 *B. subtilis* GN5_1
S2 *B. subtilis* GN5_12
S3 *B. subtilis* GN5_21
S4 *B. subtilis* GN5_23
S5 *B. subtilis* GN5_24
S6 *B. subtilis* GT12
S7 *B. subtilis* GT5_2
S8 *B. subtilis* GT5_3

V1 *B. velezensis* 0D2
V2 *B. velezensis* 0SSM31
V3 *B. velezensis* 10SSM2
V4 *B. velezensis* 12SSD3
V5 *B. velezensis* 12SSM2
V6 *B. velezensis* 7M5
V7 *B. velezensis* 7SSD1
V8 *B. velezensis* GN5_3
